# The first Japanese case of central precocious puberty with a novel *MKRN3* mutation

**DOI:** 10.1038/hgv.2017.17

**Published:** 2017-05-18

**Authors:** Junko Nishioka, Hirohito Shima, Maki Fukami, Shuichi Yatsuga, Takako Matsumoto, Kikumi Ushijima, Miyuki Kitamura, Yasutoshi Koga

**Affiliations:** 1 Department of Pediatrics and Child Health, Kurume University School of Medicine, Fukuoka, Japan; 2 Department of Molecular Endocrinology, National Research Institute for Child Health and Development, Tokyo, Japan

## Abstract

*MKRN3*, located on chromosome 15q11.2, encodes makorin ring-finger 3, which is an upstream suppressor of the hypothalamic-pituitary-gonadal axis. Mutation of this gene induces central precocious puberty (CPP). As *MKRN3* is maternally imprinted, only the paternal allele is expressed. This is the first report of an 8-year-old Japanese girl with CPP caused by a novel frameshift mutation in *MKRN3* (p.Glu229Argfs*3).

Puberty is the period during which a child develops and changes physiologically and psychologically into an adult. The timing of puberty is defined by complex interactions between environmental, nutritional, racial and genetic factors. Brain tumors, brain trauma and gene mutations, such as *KISS1* and *KISS1R* mutations, are known to cause central precocious puberty (CPP). However, in the large majority of cases, the underlying causes of CPP are unclear.

*MKRN3* encodes makorin ring-finger 3, which is thought to be an upstream suppressor of the hypothalamic-pituitary-gonadal axis. *MKRN3* mutations are known to result in CPP. As *MKRN3* is a maternally imprinted gene, only paternally inherited *MKRN3* mutations result in CPP.^1^ Indeed, maternally inherited *MKRN3* mutations do not result in CPP but rather confer a carrier status on the individual. To date, *MKRN3* mutations are the most commonly reported genetic cause of familial CPP.^1–13^ However, CPP caused by an *MKRN3* mutation has not yet been reported in Japan.

Currently, the most common medication for CPP is leuprorelin, which is a gonadotropin-releasing hormone (GnRH) analog, and the use of leuprorelin for suppressing puberty patients with CPP caused by *MKRN3* mutations has been reported. Here, we present the first report of a Japanese girl with CPP caused by a novel *MKRN3* mutation to whom leuprorelin was administered for 2 years as an effective therapy.

An 8-year-old Japanese girl, whose parents were of non-consanguineous marriage, was referred to our hospital owing to premature menarche. She was born at 39 weeks. Her thelarche began at 5 years of age. On initial examination, her pubertal stage was Tanner 3 for breast development and Tanner 2 for pubic hair growth. She exhibited growth acceleration (height; 132.3 cm (+1.1 s.d.), body weight; 40.1 kg; Figure 1) with advanced bone age (11 years old). A GnRH stimulation test showed a pubertal stage, resulting in an LH level of 2.9–52.5 U/l and an FSH level of 5.6–27.9 U/l. Her estradiol level was <25 pg/ml. Enhanced MRI showed normal findings for the pituitary. She was treated with an appropriate GnRH analog after the diagnosis of idiopathic CPP, and her pubertal development has been well controlled for 2 years.

The height of the patient’s mother was 158 cm, and her age of puberty onset was 13 years. The height of the patient’s father was 173 cm, and his age of onset of puberty was unknown. The patient’s paternal grandparents were of non-consanguineous marriage. The paternal grandfather’s height was 168 cm, and the age of puberty onset was unknown. The paternal grandmother’s height was 140 cm, and she began menarche at 10 years of age. The paternal grandmother showed precocious puberty.

This patient’s mutation was identified via mutation screening of 15 unrelated Japanese patients with CPP (10 females and five males). This study was approved by the Institutional Review Board Committee at the National Center for Child Health and Development, and was performed after obtaining written informed consent. Genomic DNA was extracted from peripheral leukocytes using standard procedures. Samples from the 15 patients with CPP were subjected to whole-exome sequencing using Nextera Rapid Capture Exome Kit (HiSeq SBS Kit v4-HS Illumina, San Diego, CA, USA) and a HiSeq2500 sequencer (Illumina). Sequence data were analyzed as described previously.^14^ In this study, we focused on 32 genes that are known to be involved in regulating the hypothalamic-pituitary-gonadal axis.^14^ Possible pathogenic mutations were confirmed by Sanger sequencing, and primer sequences are available upon request. To confirm the heterozygous mutation identified in the patient, we subcloned the PCR products, and sequenced the mutant and wild-type alleles separately. We also analyzed genomic DNA samples obtained from the patient’s family members by Sanger sequencing (Figures 2a and c).

A heterozygous 1-bp insertion in the single exon of *MKRN3* (c.683_684insA, p.Glu229fsArg*3) was found in the patient (Figure 2b). This insertion has not been reported previously, and it was not found in exome databases (The ExAC browser (http://exac.broadinstitute.org/); and the Human Genetic Variation Browser (http://www.genome.med.kyoto-u.ac.jp/SnpDB)). The patient had no pathogenic mutations in the other genes that were examined. The father and paternal grandmother of the patient were found to be heterozygous for the *MKRN3* mutation (Figure 2c), and *MKRN3* mutations were absent from the other 14 patients.

In this study, we identified the first Japanese *MKRN3* mutation causing CPP in an 8-year-old girl, who was subsequently effectively treated with leuprorelin to control her pubertal development.

In recent years, the clinical features of patients with *MKRN3* defects have gradually been determined.^1–13^

A report from Brazil showed that male patients with CPP caused by *MKRN3* mutations have a later pubertal onset than do those without *MKRN3* mutations (median age 8.2 vs.7.0 years old, respectively, *P*=0.033), and they showed typical clinical and hormonal features of CPP.^13^ By contrast, previous reports from France and Brazil revealed a median age of puberty onset of 6 years in females with CPP caused by *MKRN3* mutations.^3,11^ In particular, the report from Brazil did not find any significant associations of clinical or biological features of CPP with *MKRN3* mutations.^3^ In our female patient, thelarche began at 5 years of age, and menarche at 8 years of age, resulting in an earlier onset of puberty than in previous reports. Nonetheless, greater numbers of Japanese patients with *MKRN3* mutations are required to reveal the clinical features of CPP in the Japanese population.

The frequency of CPP with *MKRN3* mutation in Korean females is relatively low: 7 in 260 (2.7%);^7^ our investigation of female Japanese patients with CPP caused by *MKRN3* mutations revealed a relatively higher frequency of 1 in 10 (10.0%). The most recent retrospective study reporting the frequency of *MKRN3* mutations in patients with female idiopathic CPP in Brazil was 6.4%.^13^ Taken together, the frequency of female patients with CPP caused by *MKRN3* mutations in Japan is high compared with Korean and Brazilian female patients with CPP. However, the study carried out in Brazil is biased in that the patients with CPP were recruited from familial CPP groups. As the studies in Korea and Japan did not have this selection bias, the frequency of CPP caused by *MKRN3* mutations in these populations may be accurate. Our Japanese study had quite a small sample; thus, the frequency of CPP in Japanese females may decrease if more patients are recruited. Additional study is required to reveal the frequency of CPP caused by *MKRN3* mutations in Japan and worldwide.

Currently, a GnRH analog, which acts on the anterior pituitary by competing for the GnRH receptor and reducing the number of active GnRH receptors, is the gold standard treatment for CPP.^15,16^ As CPP is associated with a high risk of estrogen-dependent diseases, such as breast cancer and cardiovascular disease, GnRH analog treatment should be continued beyond the adequate age for patients with CPP caused by *MKRN3* mutations.^17^ Previous reports show that most patients with CPP caused by *MKRN3* mutation who are treated with an appropriate GnRH analog seem to have satisfactorily controlled pubertal development. For example, Macedo *et al.*^11^ reported that GnRH treatment had a therapeutic effect in six out of eight patients with CPP caused by *MKRN3* mutations. Menarche had already started when our patient was referred to our hospital at initial examination, and she was treated with the GnRH analog immediately after the diagnosis of CPP. She has been treated with leuprorelin 50 μg/kg every 4 weeks for 2 years, and her pubertal signs have not progressed, indicating that leuprorelin treatment is effective for this female Japanese patient with CPP caused by an *MKRN3* mutation.

We present here the first report of CPP in an 8-year-old Japanese girl with a novel *MKRN3* mutation and her effective treatment with leuprorelin. In this small Japanese population study, the prevalence of CPP caused by *MKRN3* mutations was moderate.

## Figures and Tables

**Figure 1:**
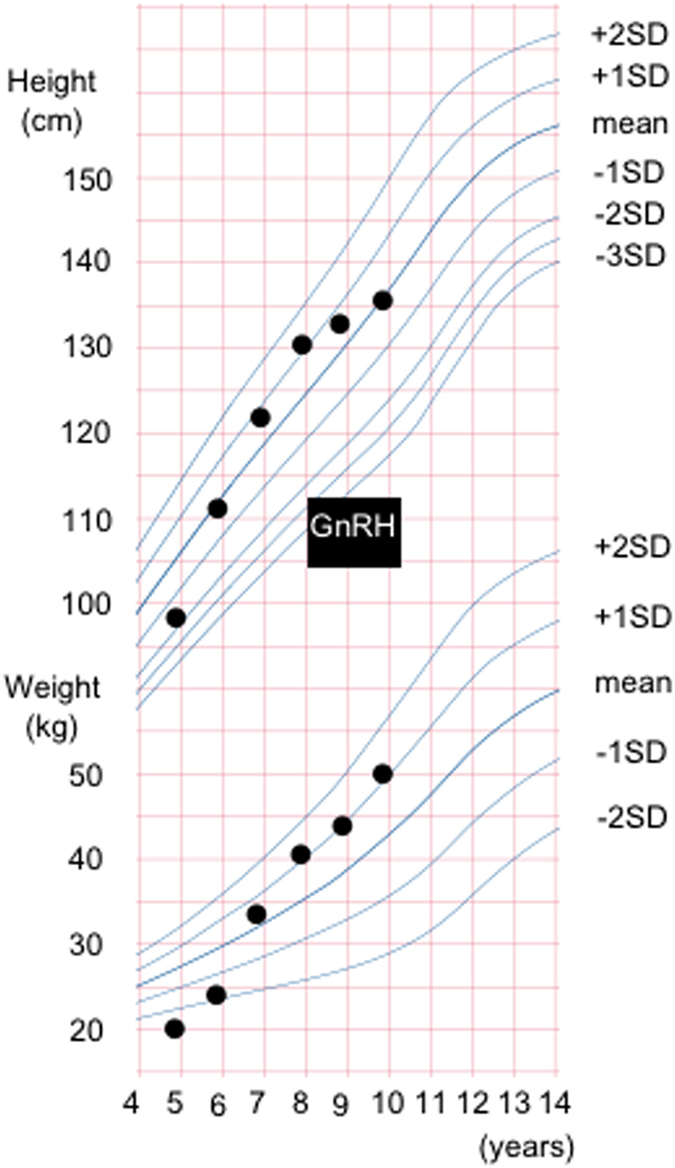
A growth curve of the patient. She has been treated with a GnRH analog for 2 years, and her CPP is well controlled. CPP, central precocious puberty; GnRH, gonadotropin-releasing hormone.

**Figure 2:**
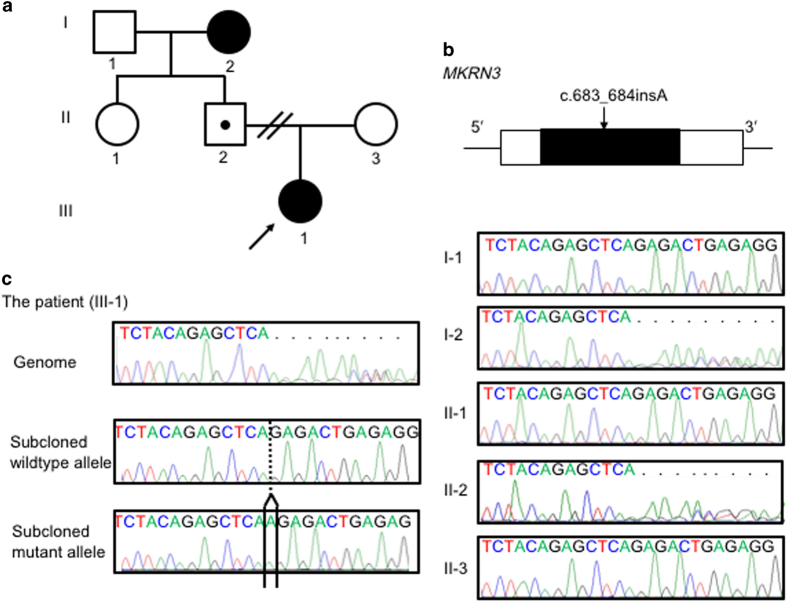
(**a**) The pedigree of this family. Squares indicate males; circles indicate females. Black symbols indicate patients with CPP. Symbols with a black point inside indicate asymptomatic carriers. White indicates patients without *MKRN3* mutations. The arrow indicates the proband. (**b**) The position of the *MKRN3* mutation in relation to genomic structure. The box indicates the exon. Black indicates the coding region; white indicates the untranslated regions. (**c**) Sanger sequencing chromatograms of the genomic region surrounding the *MKRN3* mutation. The results are shown for the patient (left) and her family members (rightl). CPP, central precocious puberty.

## References

[bib1] Abreu AP, Dauber A, Macedo DB, Noel SD, Brito VN, Gill JC et al. Central precocious puberty caused by mutations in the imprinted gene MKRN3. N Engl J Med 2013; 368: 2467–2475. 23738509 10.1056/NEJMoa1302160 PMC3808195

[bib2] Grandone A, Cantelmi G, Cirillo G, Marzuillo P, Luongo C, Miraglia del Giudice E et al. A case of familial central precocious puberty caused by a novel mutation in the makorin RING finger protein 3 gene. BMC Endocr Disord 2015; 15: 60. 26499472 10.1186/s12902-015-0056-8 PMC4619005

[bib3] Simon D, Ba I, Mekhail N, Ecosse E, Paulsen A, Zenaty D et al. Mutations in the maternally imprinted gene MKRN3 are common in familial central precocious puberty. Eur J Endocrinol 2016; 174: 1–8. 26431553 10.1530/EJE-15-0488

[bib4] Känsäkoski J, Raivio T, Juul A, Tommiska J. A missense mutation in MKRN3 in a Danish girl with central precocious puberty and her brother with early puberty. Pediatr Res 2015; 78: 709–711. 26331766 10.1038/pr.2015.159

[bib5] Neocleous V, Shammas C, Phelan MM, Nicolaou S, Phylactou LA, Skordis N. *In silico* analysis of a novel MKRN3 missense mutation in familial central precocious puberty. Clin Endocrinol (Oxf) 2016; 84: 80–84. 26173472 10.1111/cen.12854

[bib6] Abreu AP, Macedo DB, Brito VN, Kaiser UB, Latronico AC. A new pathway in the control of the initiation of puberty: the MKRN3 gene. J Mol Endocrinol 2015; 54: R131–R139. 25957321 10.1530/JME-14-0315 PMC4573396

[bib7] Lee HS, Jin HS, Shim YS, Jeong HR, Kwon E, Choi V et al. Low frequency of MKRN3 mutations in central precocious puberty among Korean girls. Horm Metab Res 2016; 48: 118–122. 25938887 10.1055/s-0035-1548938

[bib8] de Vries L, Gat-Yablonski G, Dror N, Singer A, Phillip M. A novel MKRN3 missense mutation causing familial precocious puberty. Hum Reprod 2014; 29: 2838–2843. 25316453 10.1093/humrep/deu256

[bib9] Bulcao Macedo D, Nahime Brito V, Latronico AC. New causes of central precocious puberty: the role of genetic factors. Neuroendocrinology 2014; 100: 1–8. 25116033 10.1159/000366282

[bib10] Schreiner F, Gohlke B, Hamm M, Korsch E, Woelfle J. MKRN3 mutations in familial central precocious puberty. Horm Res Paediatr 2014; 82: 122–126. 25011910 10.1159/000362815

[bib11] Macedo DB, Abreu AP, Reis AC, Montenegro LR, Dauber A, Beneduzzi D et al. Central precocious puberty that appears to be sporadic caused by paternally inherited mutations in the imprinted gene makorin ring finger 3. J Clin Endocrinol Metab 2014; 99: E1097–E1103. 24628548 10.1210/jc.2013-3126 PMC4037732

[bib12] Settas N, Dacou-Voutetakis C, Karantza M, Kanaka-Gantenbein C, Chrousos GP, Voutetakis A et al. Central precocious puberty in a girl and early puberty in her brother caused by a novel mutation in the MKRN3 gene. J Clin Endocrinol Metab 2014; 99: E647–E651. 24438377 10.1210/jc.2013-4084

[bib13] Bessa DS, Macedo DB, Brito VN, França MM, Montenegro LR, Silva MC et al. High Frequency of MKRN3 Mutations in Male Central Precocious Puberty Previously Classified as Idiopathic. Neuroendocrinology (e-pub ahead of print 26 May 2016; doi:10.1159/000446963). 10.1159/000446963 PMC5195904 27225315

[bib14] Shima H, Yatsuga S, Nakamura A, Sano S, Sasaki T, Katsumata N et al. NR0B1 frameshift mutation in a boy with idiopathic central precocious puberty. Sex Dev 2016; 10: 205–209. 27648561 10.1159/000448726

[bib15] Brito VN, Spinola-Castro AM, Kochi C, Kopacek C, Silva PC. Central precocious puberty: revisiting the diagnosis and therapeutic management. Arch Endocrinol Metab 2016; 60: 163–172. 27191050 10.1590/2359-3997000000144

[bib16] Tanaka T, Niimi H, Matsuo N, Fujieda K, Tachibana K, Ohyama K et al. Results of long-term follow-up after treatment of central precocious puberty with leuprorelin acetate: evaluation of effectiveness of treatment and recovery of gonadal function. The TAP-144-SR Japanese Study Group on Central Precocious Puberty. J Clin Endocrinol Metab 2005; 90: 1371–1376. 15598675 10.1210/jc.2004-1863

[bib17] Lakshman R, Forouhi NG, Sharp SJ, Luben R, Bingham SA, Khaw KT et al. Early age at menarche associated with cardiovascular disease and mortality. J Clin Endocrinol Metab 2009; 94: 4953–4960. 19880785 10.1210/jc.2009-1789

